# The Role of Reactive Oxygen Species in Microvascular Remodeling

**DOI:** 10.3390/ijms151223792

**Published:** 2014-12-19

**Authors:** Marius C. Staiculescu, Christopher Foote, Gerald A. Meininger, Luis A. Martinez-Lemus

**Affiliations:** Dalton Cardiovascular Research Center, and Department of Medical Pharmacology and Physiology, University of Missouri, Columbia, MO 65211, USA; E-Mails: mstaiculescu@seas.wustl.edu (M.C.S.); footec@missouri.edu (C.F.); meiningerg@missouri.edu (G.A.M.)

**Keywords:** resistance arteries, microcirculation, inward remodeling, NADPH oxidase, Nox, xanthine oxidase, mitochondria, superoxide, hypertension

## Abstract

The microcirculation is a portion of the vascular circulatory system that consists of resistance arteries, arterioles, capillaries and venules. It is the place where gases and nutrients are exchanged between blood and tissues. In addition the microcirculation is the major contributor to blood flow resistance and consequently to regulation of blood pressure. Therefore, structural remodeling of this section of the vascular tree has profound implications on cardiovascular pathophysiology. This review is focused on the role that reactive oxygen species (ROS) play on changing the structural characteristics of vessels within the microcirculation. Particular attention is given to the resistance arteries and the functional pathways that are affected by ROS in these vessels and subsequently induce vascular remodeling. The primary sources of ROS in the microcirculation are identified and the effects of ROS on other microcirculatory remodeling phenomena such as rarefaction and collateralization are briefly reviewed.

## 1. Introduction

Reactive oxygen species (ROS) such as superoxide anion and hydroxyl radical (OH^−^) are highly reactive chemical molecules that contain oxygen with unpaired electrons in their outer orbit. Within blood vessels, ROS are produced by all the cellular components of the vascular wall including endothelial cells, smooth muscle cells, fibroblasts and immune cells. Accumulating evidence indicates that ROS serve as signaling molecules that modulate acute vascular functions such as vasodilation, vasoconstriction and vascular permeability, as well as long-term vascular changes including structural remodeling of vessel segments and vascular beds [1,2,3,4]. ROS also play pivotal roles in the initiation and development of vascular dysfunctions associated with aging and diseases such as ischemic and chronic heart disease, stroke, hypertension, atherosclerosis, diabetes mellitus and chronic kidney disease [2,4,5,6,7]. An important function of ROS in the vasculature includes their capacity to modulate cellular signaling pathways that induce both acute and chronic changes in cellular phenotype. This particular function of ROS plays a key role in vascular remodeling processes through the modulation of cellular cytoskeletal properties, proliferation, migration and death, as well as, the alteration of the extracellular matrix (ECM) [1,8,9,10]. Collectively, these events are believed to be linked to changes in the mechanical characteristics of the vascular wall. This review will focus on the roles that ROS play in vascular remodeling with particular attention to the microcirculation.

The microcirculation is the most distal segment of the vascular system consisting of a network of arterioles, capillaries, and venules that are interposed between the arterial and venous systems. Its primary function is to regulate blood flow and capillary pressure that are crucial for optimizing the supply of nutrients and oxygen to tissues. In turn, these exchange processes sustain cellular metabolism and remove metabolic waste products. The flow of blood into organs is regulated by the microcirculation to meet the demands of the tissue. Within the microcirculation, the small arteries and arterioles account for the majority of the resistance to blood flow and these vessels are responsible for controlling approximately 80% of the drop in blood pressure that occurs from the large conduit arteries of the systemic circulation to the capillaries. Therefore, much emphasis has been placed on their functional and structural characteristics [11,12,13]. The resistance vessels, located proximal to capillaries, control the inflow of blood into tissues through changes in vascular diameter. As such, at any given time the diameter of a resistance vessel results from the integration of global systemic signals and local modulatory factors. Ultimately, there is a dynamic balance of vasodilatory and constrictive effects that optimize tissue perfusion, while simultaneously contributing to the regulation of systemic arterial pressure. In the microcirculation, circulating hormones, locally-derived cytokines, as well as neuronal and physical factors contribute to the level of vascular tone expressed by the resistance vessels and hence account for acute regulation of diameter.

Over the long term, resistance vessels also display adaptive properties that influence their diameter and responses to vasoactive factors. Often these longer-term adaptive changes result in the structural reorganization of the vascular wall that involves changes in both the cellular and ECM components of the vessel wall and constitutes vascular remodeling [13]. The remodeling process is achieved through a continuum of changes involving mechanisms such as cytoskeletal remodeling, cell migration, cell growth, cell proliferation, apoptosis, and ECM modifications including protein deposition, crosslinking and degradation. Each of these mechanisms is sensitive to and influenced by the oxidative environment. Changes in the oxidative environment are often associated with impairment of local blood flow and also with pathological conditions such as hypertension and diabetes.

Vascular remodeling encompasses a wide variety of structural changes occurring at all levels of the vascular tree, from large vessels down to the microcirculation. Examples include large vessel stiffening, atherogenesis, aneurisms, resistance vessel wall thickening, rarefaction or loss of small vessels and capillaries, as well as vasculogenesis and angiogenesis. In this review, our primary focus will be to provide an overview of the relationship between ROS production and the different mechanisms of remodeling processes that occur at the level of the microcirculation. First, we will discuss some of the most prominent enzymatic systems involved in the production of ROS in the vasculature. Then we will discuss the mechanisms of remodeling found primarily in resistance arteries and arterioles, and how they are affected by ROS. Where needed, we will draw upon information obtained from large vessel studies and cell-based *in vitro* experiments that provide insight on the mechanisms that modulate microvascular structure. Vascular remodeling of the resistance arteries will be described using the definitions introduced by Mulvany *et al*. [14]. Consequently, vascular remodeling will be classified based on the changes observed in the passive luminal diameter of vessels and the cross-sectional area of the vascular wall. The remodeling is inward or outward depending on whether the passive luminal diameter decreases or increases, and hypotrophic, eutrophic or hypertrophic if the amount of wall material decreases, remains constant or increases, respectively. We will also briefly cover the role of ROS in rarefaction of the capillary bed and angiogenesis. 

## 2. The Remodeling Process

Vascular remodeling processes involve intracellular and extracellular modifications that change the functional relationship between cells, and between cell and the ECM components of the vessel wall. This results in alterations in vascular function and performance [13,15]. Vascular remodeling may be induced by an array of factors, such as mechanical and hemodynamic forces, as well as neurohumoral or paracrine agents. In addition, multiple pathological conditions are associated with remodeling of the microcirculation, of which hypertension has received considerable attention due to its high incidence and contribution to cardiovascular mortality [16]. Several studies indicate that inward eutrophic and hypertrophic remodeling are the two major remodeling types encountered in the resistance vessels of humans and animal models of hypertension [17,18,19,20,21,22,23,24,25,26]. Inward eutrophic remodeling is believed to occur as vessel wall components rearrange around a smaller luminal diameter without synthesis of new materials or degradation of existing constituents [13]. Hypertrophic remodeling involves an increase in cross-sectional area of the vascular wall that includes cell proliferation and alterations in the amount, composition and arrangement of ECM components such as elastin, fibronectin and collagen [27]. Inward eutrophic remodeling is the most common structural change encountered in the resistance vessels of patients with essential hypertension, as well as in those of spontaneously hypertensive rats (SHRs) [28,29,30]. In secondary forms of hypertension, resistance artery and arteriolar remodeling is inward hypertrophic [27,31,32]. This hypertrophic remodeling has also been observed in hypertensive patients with type 2 diabetes [33,34], and in patients with acromegalia [25] and Cushing syndrome [24]. This suggests that the mechanisms contributing to remodeling can be differently modulated depending on background factors associated with the pathological conditions.

In contrast to hypertension, where inward remodeling of resistance arteries is most prevalent, outward remodeling occurs in vessels with chronically increased blood flow [35]. Flow-induced outward remodeling is believed to be an important physiological adaptation to increased metabolic demands, such as those encountered during exercise training or pregnancy [36,37,38,39]. The main trigger for vessels to remodel outwardly is an increased level of shear stress associated with the augmented flow of blood. During the prolonged increase in shear stress the endothelium releases factors that induce vasodilation and are believed to initiate a structural increase in vascular diameter accompanied by hypertrophy of the media. This outward hypertrophic remodeling results in normalization of shear stress and wall circumferential stress.

Mechanistically, the explanation for the inward or outward remodeling processes observed in resistance vessels may reside in the balance of stimuli that support vasodilation or vasoconstriction. It has been suggested that the intrinsic ability of resistance vessels to effectively control their diameter through the myogenic response plays a role in directing the overall characteristics of the remodeling process [30,40]. The myogenic response is defined as the ability of resistance vessels to constrict or relax in response to increases or decreases in intravascular pressure, respectively [41]. This local pressure-dependent mechanism is believed to help resistance arteries normalize circumferential stress, and during prolonged vasoconstriction to allow for the rearrangement of the cellular and extracellular components of the vascular wall leading to inward eutrophic remodeling [13,42]. When the myogenic response is not sufficient to normalize circumferential stress, the vessel undergoes hypertrophy as a compensatory mechanism [42]. The ultimate purpose of these adaptive changes in the structural diameter of resistance vessel is thought to play a role in protecting the fragile capillaries from pressure overload and ultimately prevent end-organ damage due to changes in perfusion and pressure [17]. The above postulates are supported by a growing body of evidence suggesting that the structure of resistance vessels is plastic and dynamic, with all components of the vessel wall, cellular and extracellular, continually changing to regulate the structural state of the vessel in order to maintain optimal conditions for vasodilation and vasoconstriction [13,15,43,44]. While all determinants of the remodeling process are not yet fully elucidated, current evidence suggests that alterations in intracellular cytoskeletal structure, cell attachment, cell migration, growth and apoptosis as well as ECM reorganization, synthesis and degradation occur to varying degrees to support the transition from prolonged vasodilation or vasoconstriction to the remodeled vascular state [13].

## 3. Sources of Reactive Oxygen Species in the Microcirculation

### 3.1. NADPH Oxidases

The nicotinamide-adenine-dinucleotide phosphate (NADPH) oxidases (Noxs) are membrane bound enzymes that transfer electrons from NADPH to molecular oxygen, thus generating NADP+, superoxide and other downstream ROS [45]. There are seven members of the Nox family of Noxs: Nox1, Nox3, Nox4, Nox5, Duox1, Duox2 and the prototypic NADPH oxidase Nox2 (gp91phox). The distribution of the different Nox family members is quite heterogeneous across different tissues. Nox1 is found in colon, prostate, uterus and vascular cells, and its function(s) is mainly related to cell growth [46]. Nox2 is the phagocytic Nox and is widely distributed across tissues [45]. In contrast, Nox3 is found almost exclusively in the inner ear [47]. Nox4 is a widely distributed enzyme abundant in kidney, pancreas, placenta, ovary, testis, skeletal muscle and vascular cells [48,49]. It is for the most part constitutively expressed, but its precise physiological function is not well known. Nox5 is a calcium-dependent enzyme, not found in mice and rats, that is primarily expressed in lymphoid tissues and testis [49]. Duox1 and Duox2 are both found in the thyroid, where they are involved in thyroid hormone synthesis, and in epithelia of the lung and gastrointestinal glandular tissues where they possibly serve in host defense [50,51]. The Noxs are a primary source of superoxide anion in the vasculature. Nox1, Nox2, Nox4, and Nox5 have been identified in blood vessels and their roles have been extensively reviewed elsewhere [52]. It is also apparent that the expression of these specific Noxs varies in different vascular beds, cell types within blood vessels, and even subcellular compartments [53].

The prototypical phagocytic NADPH is composed of five subunits: p47phox, p40phox, p67phox, p22phox and the catalytic subunit gp91phox [54]. Two of the units reside in the membrane, gp91phox and p22phox, where they form a heterodimeric flavoprotein (cytocrome b558). The other three subunits reside in the cytosol. Upon stimulation, p47phox is phosphorylated and the cytosolic units form a complex that includes the small guanosine triphosphate (GTP)ase Rac [55]. This complex is translocated to the membrane where it interacts with gp91phox and p22phox. This interaction activates the enzyme resulting in superoxide anion production. The regulation of Nox activity is intricate, involving different signaling pathways. In addition, the ability of the different Noxs to produce superoxide varies depending on the characteristic of the supportive proteins. In general, Nox1, and 2 require Rac to initiate or enhance their production of superoxide, whereas Nox5, Duox1 and Duox2 do not require Rac [55,56]. Nox4 does not contain any Rac binding sites but its activity may be modulated by Rac1 [9,56]. The role of Rac on the activity of the Noxs also depends on whether the Nox supportive proteins in the cell are p47phox and p67phox or their homologues Noxo1 and Noxa1, respectively. It also appears that different Noxs have different affinities for Rac1 or Rac2 [55], but the overall effect of either Rac on the activity of the Noxs likely depends on the predominant Rac expressed in each specific cell or tissue.

Additional levels of Nox-activity regulation are provided by different enzymes and signaling pathways within the cell. For example, in vascular smooth muscle cells (VSMCs) Nox activity is regulated by protein disulfide isomerase, a chaperone enzyme involved in regulating the redox status of the cell and the processing of proteins [57]. Also in VSMCs and in neutrophils, Nox1 and 2 have been shown to be regulated by the chloride/proton antiporter CIC-3 [58,59]. The enzyme polymerase delta-interacting protein or Poldip2 has also been shown to modulate the activity of Nox4 in VSMCs [60]. Interestingly, Poldip2 is involved in the strengthening of focal adhesions and the formation of stress fibers via Rho dependent pathways [60,61]. This is important in the context of vascular remodeling, as we have shown that vasoconstriction-induced inward remodeling of resistance vessels requires ROS and actin polymerization [15,62]. Furthermore, the transcription and activation of Nox enzymes are induced by a wide variety of stimuli such as G-protein coupled receptor agonists (angiotensin II, serotonin, thrombin, endothelin-1), mechanical stimuli (shear stress, pressure, stretch), growth factors (transforming growth factor β, epidermal growth factor, platelet derived growth factor), and inflammatory cytokines (interleukins, tumor necrosis factor α), most of which have been associated with vascular remodeling processes [9,63,64,65,66].

In the vascular system, increased Nox activity and expression have been described in a number of physiological and pathological conditions associated with vascular remodeling. For example, hypertension in general has been associated with an augmented production of superoxide by Nox in conduit vessels. Nox activity is increased in SHR rats [67], deoxycorticosterone acetate (DOCA) salt sensitive rats [68] or mice [69], and angiotensin II-infused hypertensive rats [70,71]. At the level of the microcirculation, increased activity of Nox has been reported in the coronary and mesenteric arterioles of SHR rats [72]. In addition, it has been reported that in SHRs treated with testosterone addition of the antioxidant and non-specific Nox inhibitor, apocynin, results in decreased migration of VSMCs [73]. In hypertension, angiotensin II appears to be an important player in the activation of Nox and the vascular remodeling process. Angiotensin II infusion induces an increase in Nox activity and remodeling of the mesenteric [74,75] and renal microvasculature [76]. Conversely, the vascular remodeling and increase in Nox activity induced by angiotensin II is diminished in mice treated with apocynin [77]. In addition, treatment with angiotensin-converting enzyme (ACE) inhibitors or angiotensin II receptor blockers results in reduced expression of p22phox, p47phox, gp91phox and reverses the cardiovascular remodeling observed in the SHR [78]. Furthermore, a recent study reported that treatment with atorvastatin down-regulates angiotensin II-induced Nox1 expression and reduces vascular remodeling in rats through a putative antioxidant effect [79].

The activity and expression of the Noxs have also been reported to play important roles in the regulation of angiogenesis. For example, in the angiogenic response to hypoxia-reoxygenation in the heart, the expression of the proangiogenic factor vascular endothelial growth factor (VEGF) is reduced by the pharmacological blockade of Nox or the transcriptional knockdown of the NADPH oxidase subunit p47phox [80]. Similarly, angiopoetin-1-mediated angiogenesis is dependent on the activity of Nox and the presence of H_2_O_2_ [81]. A detailed description of the role of ROS and Nox on angiogenesis is beyond the scope of this review, and the reader is referred to several other publications [82,83,84]. Overall, the accumulated evidence supports the concept that alterations in expression and activity of the Nox enzymes are associated with changes in microvascular structure and function.

### 3.2. Nitric Oxide Synthase

**Figure 1:**
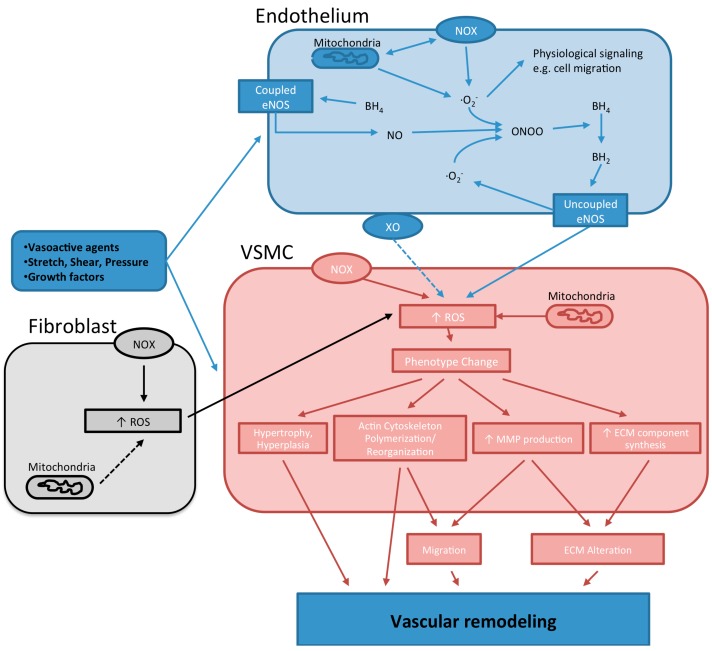
Reactive oxygen species (ROS)*-*dependent mechanisms underlying vascular remodeling. Various stimuli activate ROS-generating enzymes located either in the endothelium or the vascular smooth muscle cells (VSMCs). In the endothelium, activation of Nox results in the production of superoxide and may also induce an increased release of superoxide from the mitochondria. The superoxide anion produced can activate physiological signaling pathways and/or interact with nitric oxide (NO) to produce peroxynitrite (ONOO). The latter scenario is particularly important when superoxide is produced in excess or when ROS scavenging molecules are insufficient. Peroxynitrite interacts with tetrahydrobiopterin (BH_4_) decreasing its availability as a precursor for the synthesis of NO. As a result, endothelial nitric oxide synthase (eNOS) becomes uncoupled and starts to produce superoxide. The superoxide, in turn, interacts with NO to produce ONOO, further reducing the availability of BH_4,_ thus promoting eNOS uncoupling. In VSMCs, exogenous or endogenous ROS induce phenotype changes. These alterations in phenotype are associated with changes in cellular growth and apoptosis that result in the hypertrophy and hyperplasia of VSMCs. Furthermore, VSMCs also reorganize their actin cytoskeleton, increase the production of extracellular matrix (ECM) components and the activity of matrix metalloproteinases (MMPs). The results of these phenotypical changes are increased VSMC proliferation, migration/repositioning and the reorganization of the ECM. These processes lead to changes in the micro architecture of the vascular wall and changes in vessel diameter that underlie vascular remodeling. Dashed arrows represent pathways that are not confirmed.

Endothelial nitric oxide synthase (eNOS), one of the three isoforms of NO synthases, uses l-arginine and molecular oxygen to produce l-citrulline and NO. Functionally eNOS is a homo-dimer enzyme that binds calmodulin in the presence of Ca^2+^ to facilitate the production of NO. The enzyme requires the presence of cofactors such as flavin adenine dinucleotide (FAD), flavin mononucleotide (FMN), and (6*R*-)5,6,7,8-tetrahydrobiopterin (BH_4_). The synthesis of NO by eNOS requires the hydroxylation of l-arginine to *N*^ω^-hydroxy-l-arginine that is then oxidized to NO and l-citrulline. Nitric oxide causes vascular dilation and participates in blood pressure control. It also has anti-atherosclerotic and vasoprotective effects by interfering with coagulation [85], leukocyte adhesion [86,87,88] and smooth muscle cell proliferation [89]. However, in certain circumstances such as those in which BH_4_ or l-arginine are deficient, eNOS transfers electrons form NADPH directly to molecular oxygen, resulting in the production of superoxide anion instead of NO, a process known as eNOS uncoupling (Figure 1). The uncoupling of eNOS, in a rodent model of metabolic syndrome, has been associated with increased production of superoxide and hypertrophic remodeling of resistance arteries [90].

A number of conditions can result in a reduced availability of l-arginine that uncouples eNOS. Endothelial cells express arginase, an enzyme that uses l-arginine as a substrate to produce l-ornithine and urea. There are two arginase isoforms; arginase I is located in the cytoplasm and mainly found in the liver, while arginase II is located in the mitochondria and has a wide tissue distribution with the highest levels of expression in the kidney and prostate. In certain pathological conditions, arginase I and II become highly expressed and decrease the availability of l-arginine leading to eNOS uncoupling and an increased production of ROS. Results obtained in bovine aortic endothelial cells indicate that peroxynitrite and H_2_O_2_ increase arginase activity and lead to increased production of superoxide anion [91]. The activity and expression of arginase appears to be increased through a protein kinase C (PKC) mediated activation of the Rho/Rho kinase (ROCK) pathway. Furthermore, a recent study found that in the aorta of aged mice there is an increase in arginase II activity that causes eNOS uncoupling and impairs endothelial mediated vasorelaxation [92]. In those mice, siRNA treatment targeting arginase II markedly improved acetylcholine dependent vasodilation indicating that arginase downregulation increased the availability of l-arginine for eNOS. Thus, current data suggest that a major factor causing eNOS uncoupling is decreased l-arginine availability as a consequence of increased arginase enzyme activity.

Deficiency of BH_4_ is another factor that promotes eNOS uncoupling (Figure 1). l-citrulline and NO production by eNOS, in endothelial cells, is closely related to the intracellular levels of BH_4_ [93], which in turn are dependent upon the fine balance between its synthesis and degradation. Decreased levels of BH_4_ have been found in DOCA-salt hypertensive rats [94], in the aorta of insulin resistant rats [95], in the plasma of SHRs [96], as well as in patients with diabetes and essential hypertension [97,98]. In all those examples supplementation of BH_4 _enhanced vasodilation and had an overall beneficial effect on endothelial function. BH_4_ is one of the most potent naturally occurring reducing agents and is very susceptible to oxidation. It has been proposed that in the initial stages of hypertension, Nox activation leads to the production of superoxide anion that, in turn, reacts with NO producing peroxynitrite and uncoupling eNOS [94]. Peroxynitrite, even at small concentrations, oxidizes BH_4_ and therefore plays a major role in the uncoupling of eNOS [99,100]. Through a feed-forward mechanism, the production of peroxynitrite continues to amplify eNOS uncoupling and the consumption of BH_4_. In addition, a diminished biosynthesis of BH_4_ may further exacerbate eNOS uncoupling in a number of pathological conditions where BH_4_ is being oxidized. For example, the overexpression of GTP cyclohydrolase I (GTPCH I), a rate limiting enzyme in the synthesis of BH_4_, has been shown to abrogate superoxide production in mesenteric arteries of DOCA-salt treated mice [101]. In that study, GTPCH I overexpression also reduced microvascular hypertrophic remodeling and attenuated hypertension. Another mechanism that contributes to BH_4_ depletion and eNOS uncoupling involves the attenuated recycling of 7,8-dihydrobiopterin (BH_2_), the oxidized form of BH_4_ [102] (Figure 1). For example, it has been reported that in endothelial cells treated with angiotensin II, BH_4_ deficiency is the result of diminished dihydrofolate reductase (DHFR) activity, the enzyme responsible for BH_2_ to BH_4_ recycling. In those cells, downregulation of DHFR was caused by H_2_O_2_ produced as a result of angiotensin II-mediated activation of Nox [103]. Similarly, in DOCA salt-treated mice the increased production of ROS by uncoupled eNOS is markedly reduced in p47phox-knockout DOCA mice, suggesting that Nox contributes to the uncoupling of eNOS in that model of hypertension [94]. In support for a role of the Noxs in diminishing the recycling of BH_2_ to BH_4_ by DHFR, a recent study found that Nox1 is involved in the uncoupling of eNOS and the downregulation of DHFR in diabetic mice [104]. In that study the overexpression of DHFR or the administration of folic acid resulted in improved endothelial function and recoupling of eNOS. All this evidence suggests that the depletion of BH_4 _represents an important pathological mechanism leading to eNOS uncoupling and increased production of ROS in vascular diseases associated with vessel remodeling.

### 3.3. Xanthine Oxidase

Xanthine oxidase (XO) is another vascular source of ROS that is expressed mainly in the endothelium. XO is one of the two isoforms of xanthine oxidoreductase, the other isoform being xanthine dehydrogenase. Xanthine dehydrogenase produces NADH and uric acid from hypoxanthine or xanthine, while XO produces superoxide and H_2_O_2_ by transferring an electron from xanthine or hypoxanthine to molecular oxygen [105,106]. Xanthine dehydrogenase can be converted to XO by the reversible oxidation of cysteine residues, or it can be irreversibly transformed into XO through proteolysis [107,108,109]. XO produces a mix of H_2_O_2_ and superoxide (with H_2_O_2 _being the main ROS) depending on the concentration of oxygen in tissues, cellular localization of the enzyme or availability of substrates [110]. XO expression and activity are increased by angiotensin II in patients with coronary disease. Its activation is ROS dependent and is inhibited by allopurinol and losartan (an angiotensin II receptor blocker) [111]. Furthermore, oscillatory shear stress triggers Nox dependent XO activation and release of superoxide [112], an important contributor to vascular injury and remodeling. *In vitro*, XO activation promotes endothelial cell survival and stimulates angiogenesis through the release of VEGF [113]. In contrast, XO-derived superoxide, by promoting endothelial dysfunction, contributes to hypoxia-induced pulmonary hypertension and remodeling [114]. In some models of hypertension such as the SHR and Dahl-salt sensitive rat, inhibition of XO reduces the production of ROS and lowers blood pressure [115,116]. In contrast, XO appears not to be an important contributor to the elevated blood pressure observed in glucocorticoid-induced hypertension [117]. Based on these conflicting data, the role of XO in hypertension, vascular injury and microvascular remodeling remains to be clearly defined. 

### 3.4. Mitochondrial Electron Transport System

The mitochondrial electron transport chain is one of the major sources of ROS in mammalian cells. Superoxide is generated as a byproduct of ATP production in the process of oxidative phosphorylation. During the transfer of electrons from NADH to molecular oxygen in the mitochondria some superoxide is produced, which can escape into the cytosol via anion channels [118,119,120]. In addition, the electron transport chain may become uncoupled during episodes of tissue ischemia or cellular hypoxia resulting in increased production of superoxide. Elevated mitochondrial production of ROS has been associated with an increased production of VEGF and angiogenesis [121]. However, the specific action of mitochondrial ROS on the vasculature may vary with vascular bed and the amount of ROS produced. In pulmonary arteries, hypoxia induces Nox activation, increases ROS production and the contractility of smooth muscle cells. These hypoxic effects can be reduced by rotenone, an inhibitor of mitochondrial complex I, as well as inhibitors of PKCε [122], suggesting that mitochondrial uncoupling participates in the process. In DOCA-salt sensitive rats it has been reported that the use of inhibitors of mitochondrial electron transport complexes II and IV prevent the increase in ROS associated with hypertension [123]. Further evidence on the contribution of mitochondrial ROS to hypertension comes from experiments showing that aldehyde dehydrogenase 2, a mitochondrial enzyme, diminishes the contractile effects of angiotensin II in hypertensive mice by preventing ROS generation [124]. Recently, a positive feedback mechanism has been proposed for the increase in mitochondrial ROS production induced by angiotensin II at the level of the endothelium [125]. In the proposed mechanism, angiotensin II triggers Nox activation, followed by the activation of mitochondrial K_ATP_ channels resulting in the depolarization of the mitochondria and ROS production. The increase in cytosolic ROS results in PKC activation that further increases Nox activity. In this paradigm, the mitochondria behave as an amplifier for ROS in a positive feedback loop, which would eventually result in uncoupling of eNOS, endothelial dysfunction and impaired vascular relaxation (Figure 1). In regard to vascular remodeling, a role for mitochondria in the process has been suggested by experiments showing that in a model of vascular injury a reduction in superoxide dismutase-2 (SOD2, MnSOD) activity results in an increased proliferation and migration of neointimal cells [126]. Overall, substantial evidence implies that mitochondrial ROS contributes to vessel remodeling, angiogenesis and blood pressure regulation, but additional work is needed to elucidate the exact mechanisms promoting and regulating mitochondrial ROS production and its effects on vascular remodeling.

## 4. The Role of ROS in Vascular Remodeling

### 4.1. ROS in Remodeling of the Microcirculation 

ROS serve a physiological role in the vasculature and contribute as secondary messengers in adventitial fibroblasts, VSMCs, and endothelial cells. Overall, increases in the bioavailability of vascular ROS can stimulate collagen deposition, alter the activity of matrix metalloproteinases (MMPs), and promote the rearrangement of the cytoskeleton, leading to cell migration, growth or apoptosis [127] (Figure 1). All these processes have clear repercussions on the structure of the vascular wall, suggesting that ROS play important roles in microvascular remodeling. However, only a small number of studies have directly investigated the involvement of ROS on the structural modification of microvessels (Table 1). *In vivo*, studies have shown that the inward and hypertrophic microvascular remodeling processes that are observed in animal models of hypertension are decreased by blockade of the angiotensin II type 1 receptor [128], up-regulation of SOD [76] or treatment with the SOD mimetic, tempol [62,76,129,130]. *Ex* *vivo*, we have shown that incubation of isolated rat cremaster arterioles with tempol or apocynin prevents the inward remodeling induced by prolonged agonist-induced vasoconstriction [62]. Touyz and Schiffrin [131] also showed that VSMCs isolated from arterioles of hypertensive patients produce greater amounts of ROS than normotensive controls in response to angiotensin II stimulation. These results suggest that superoxide is an important ROS associated with the pathological microvascular remodeling observed in hypertension. They also suggest that ROS-dependent signaling by angiotensin II is an important mechanism associated with the inward and hypertrophic types of resistance vessel remodeling in hypertension.

**Table 1:** Representative studies showing ROS contribution to remodeling of resistance arteries.

Vascular Bed	Experimental System	Stimulus	ROS Species	Type of Remodeling	ROS Inhibitor	Ref.
Mouse mesenteric arteriole	PPARγ KO mice	angiotensin II	superoxide, reduced SOD3 expression	Eutrophic remodeling, Hypertrophic remodeling	-	[75]
Human subcutaneous arteriole	Human	Cushing syndrome	superoxide	Hypertrophic remodeling	-	[24]
Mouse mesenteric arteriole	(NZO) mice	-	superoxide, peroxynitrite	Hypertrophic remodeling	Tempol	[90]
Rat mesenteric arteriole	Wistar rats (female) ovareiectomized	high flow	superoxide	Hypertrophic remodeling	-	[39]
Rat mesenteric arteriole	Zucker rats	high flow, hyperglycemia	superoxide	Hypertrophic remodeling	Tempol	[132]
Mouse mesenteric arteriole	BALB/c male mice	angiotensin II	superoxide	Hypertrophic remodeling	Apocynin	[77]
Rat mesenteric arteriole	Wistar rats	angiotensin II	superoxide	Inward eutrophic remodeling	Atorvastatin **	[79]
Mouse basilary artery	PPAR-gamma KO mice	-	superoxide	Inward hypertrophic remodeling	Tempol	[32]
Rat cremasteric arteriole	Sprague-Dawley rat	norepinephrine, angiotensin II	superoxide, hydrogen peroxide	Inward remodeling	Tempol, Apocynin	[62]
Rat middle cerebral artery	SPSHR rats	serotonin	superoxide	Inward remodeling	Tempol	[130]
Rat mesenteric arteriole	Wistar rats	low flow	superoxide	Inward remodeling	Tempol, Apocynin	[133]
Rat mesenteric arteriole	Sprague-Dawley rat	angiotensin II	superoxide	Inward remodeling	-	[71]
Mouse aferent arteriole	SOD1 tg, SOD1 KO mice	angiotensin II	superoxide	Inward remodeling	Tempol	[76]
Rat middle cerebral artery, basilary artery	SHR	-	superoxide	Inward remodeling, Hypertrophic remodeling	Telmisartan ^#^ (ARB)	[128]
Rat mesenteric arteriole	Wistar rats	low flow, high flow	superoxide	Inward remodeling, Outward remodeling	Tempol	[134]
Rat/Mouse mesenteric arteriole	Wistar rats, eNOS KO mice	low flow, high flow	superoxide, hydrogen peroxide	Inward remodeling, Outward remodeling	Apocynin, Catalase	[135]
Rat mesenteric arteriole	Wistar rats	high flow	superoxide	Outward hypertrophic remodeling	Tempol, Perindopril *, Candesartan ^#^	[136]
Rat mesenteric arteriole	Zucker rats	high flow	superoxide	Outward hypertrophic remodeling	Tempol, Catalase, SOD	[137]
Rat mesenteric arteriole	Wistar rats	high flow	superoxide	Outward remodeling	Tempol, Apocynin	[138]

PPARγ, KO-Peroxisome proliferator-activator receptor; NZO-New, Zealand obese; SHR, Spontaneously hypertensive rats; SPSHR, Stroke prone spontaneously hypertensive rats; SOD1 tg, Superoxide dismutase 1 transgenic; SOD1 KO, Superoxide dismutase 1 knock out; ^#^ Angiotensin II receptor blocker; * Angiotensin-converting-enzyme inhibitor; ** Statin.

Angiotensin II increases ROS production and signaling by stimulating the activity of Noxs and by activating redox-sensitive genes [139,140,141]. Although angiotensin II is usually associated with vasoconstriction and hypertension, ROS and angiotensin II-dependent signaling are also associated with the outward remodeling induced by high-flow conditions in mesenteric resistance arteries [136,138]. Multiple studies have shown the effects of high or low flow conditions in resistance arteries [39,133,134,135,142,143]. High flow induces outward hypertrophic remodeling, while low flow induces inward hypotrophic remodeling. Interestingly, both remodeling processes are associated with angiotensin II-dependent signaling and an increased production of ROS by the vessel wall. The proportion of the increase in ROS that is dependent on angiotensin II remains to be fully determined, but reports indicate that the specific effects that ROS and angiotensin II have on the vascular wall during high or low flow conditions are different. It appears that superoxide mediates the luminal enlargement effect of high flow, while angiotensin II mediates the hypertrophic effect through extracellular-signal-regulated kinases (ERK)1/2 activation [136]. In contrast, angiotensin II-dependent constriction appears to mediate low flow-induced inward remodeling with only a marginal effect of superoxide on the cross-sectional area of the wall [133]. It is believed that hemodynamics and the local signaling environment, in particular the local production of vasodilators and vasoconstrictors, affect the contractile and synthetic state of cells within the vascular wall and thus guide the type of remodeling [142]. This is consistent with the fact that multiple types of vascular remodeling can coexist in a vascular bed or in the same individual in different vascular beds. The effects of high or low flow conditions on resistance artery remodeling highlight the role that the contractile state of the vasculature has on the type of remodeling. High flow is associated with vasodilation and low flow with vasoconstriction in concert with increased or decreased production of NO, respectively [35]. In both the inward and outward remodeling processes, it appears that ROS participate in part via their capacity to activate MMPs [35,62,144,145]. The specific vascular cells, signaling cascades, and ROS molecules involved in each type of remodeling, however, are not yet fully determined. Furthermore, pathological states such as those encountered in obesity and diabetes modulate the effects that ROS have on the remodeling process. For example, mesenteric arteries from obese Zucker rats develop outward remodeling when exposed to high flow. The presence of diabetes in those rats reduces the high-flow dependent outward remodeling despite the presence of increased ROS production [132,137,146,147].

### 4.2. Reactive Oxygen Species and the Phenotype of Vascular Smooth Muscle Cells

An important aspect of the vascular remodeling processes relies on the capacity of VSMCs to actively contract the vessel, produce ECM proteins, degrade those proteins, change position within the vascular wall and/or proliferate. VSMCs can perform both contractile and synthetic functions. These functions are associated with specific cellular morphologies and the cellular expression of different marker proteins, as well as proliferative and migratory capabilities. The particular phenotype VSMCs adopt depends on a wide array of environmental cues, such as physical factors (stretch and shear stress), biochemical factors, and biophysical signals contained within neighboring ECM components. Within the adult vessel wall, VSMCs are generally believed to exist in a low proliferative state with reduced synthetic activity known as the contractile phenotype. Some of the most relevant markers present in VSMCs with the contractile phenotype are smooth muscle myosin heavy chain, smoothelin, and desmin. However, during development or in pathological conditions such as those found in vascular injury or atherosclerosis, VSMCs lose those contractile marker proteins [148]. This results in a change to a more synthetic phenotype, characterized by increased VSMC migration and proliferation, hypertrophy, and ECM protein deposition [148]. In hypertension, the role of VSMC phenotype switching is evidenced by the enhanced proliferation of cultured VSMCs from SHRs [149,150] and the expression of genes associated with the synthetic phenotype, such as cellular retinol binding protein (CREB-1). Also, in stroke-prone SHRs, the presence of brain lesions is associated with the expression of markers specific for the synthetic phonotype in VSMCs, such as non-muscle myosin heavy chain [151]. Furthermore, in patients with essential hypertension that develop preeclampsia a reduction in VSMC contractile markers has been shown to predict the persistence of hypertension after delivery [152]. Similarly, reduced smooth muscle contractile markers (α-smooth muscle actin, desmin, smooth muscle myosin heavy chain) were found in patients with pulmonary hypertension [153]. Although the mechanisms involved in the development of a particular VSMC phenotype are not yet fully determined, a series of physical and biochemical factors have been proposed as contributors to this phenotypic modulation. Growth factors, such as platelet-derived growth factor-BB (PDGF-BB) and transforming growth factor-β1 (TGF-β1), or modulators of tone such as NO, angiotensin II and endothelin-1, in addition to mechanical stimulation of vessels, have been shown to induce phenotypical changes in VSMCs [148,154]. Many of these factors also promote the production of ROS, suggesting that ROS may be involved in the phenotypic modulation of VSMCs. Whether such phenotypical changes occur at the level of the resistance arteries in the microcirculation remains to be fully elucidated. Our previous finding—that a number of VSMCs reposition during the remodeling induced by prolonged exposure to vasoconstrictor agonists in isolated arterioles—suggests that VSMCs may change phenotype rapidly during the inward remodeling process [155]. However, whether this represents a full phenotypic change from a contractile to a synthetic phenotype is controversial. It could be a normal property of VSMCs, especially when one considers that the remodeling observed in hypertension at the level of the resistance arteries is for the most part eutrophic; that is, there is no hypertrophy or hyperplasia of VSMCs.

The overall role of ROS in VSMC phenotypic modulation is not fully understood. However, a number of studies clearly indicate that ROS are involved in the process. Studies in atherosclerosis have shown that superoxide induces the synthetic phenotype as well as cytoskeletal changes in VSMCs [156,157]. Furthermore, superoxide anion (in conjunction with H_2_O_2_) increases PDGF [158,159] and collagen production in VSMCs [160], which, as mentioned above, are characteristic of the synthetic phenotype. In animal models of vascular injury, it has been shown that superoxide increases VSMC migration leading to vascular remodeling of conduit arteries [126,161]. A similar role has been found for H_2_O_2_, which enhances hypertrophy, proliferation, and migration of VSMCs in culture [162]. However, the role of H_2_O_2_ on inducing the synthetic phenotype in VSMCs is controversial, and it has been shown that in cultured human VSMCs the production of H_2_O_2_ is necessary to maintain the contractile phenotype [163]. The specific location of ROS production within the cell may also be important, as it is believed that in VSMCs, Nox1 and Nox4 may have opposite effects due in part to their subcellular localization [164,165]. In addition, Nox4 is constitutively active and appears to produce mostly H_2_O_2_, while Nox1 produces mostly superoxide. It also appears that the local environment plays a role on the effect that specific ROS have on modulating the VSMC phenotype. For example in pulmonary arterial smooth muscle cells, ROS produced by Nox4 promote proliferation [166], whereas Nox4 appears to maintain the contractile phenotype of VSMCs from the systemic circulation [167]. Although little is known about the role that ROS play on the phenotypic modulation of VSMCs in resistance vessels, it is likely that ROS have functions similar to those observed in conduit arteries. In addition, it has been shown that endothelial dysfunction in resistance arteries is associated with low production of NO and increased production of ROS. NO is known to maintain VSMCs in a low proliferative state [168]; thus a dysfunction in the production of NO might result in phenotypic changes in VSMCs. However, it remains to be determined if it is only the reduction of NO, the increase in ROS, or both that change the proliferative capacity and the phenotype of VSMCs. As the origin and characteristics of VSMCs vary along the vascular tree [169] and possibly within the same vessel, further investigation is needed on the exact role of ROS-induced VSMC phenotypic modulation at the level of the microcirculation in different vascular beds. 

### 4.3. ROS-Induced Vascular Smooth Muscle Cell Migration

The vascular remodeling process, especially that associated with vascular injury, involves the migration of VSMCs. Induction of VSMC migration is mediated by a wide range of signaling molecules, such as angiotensin II, PDGF, VEGF, thrombin, arachidonic acid, and norepinephrine [141,170,171,172,173,174,175]. Most of these molecules also generate ROS, and the involvement of ROS in VSMC migration is well documented in neointimal formation and vessel injury repair [176]. A few studies have also looked at the migration of VSMCs in animal models of hypertension and diabetes [73,177,178,179,180,181,182,183]. *In vitro*, a seminal study detailing the importance of ROS in VSMC migration, determined that H_2_O_2_ is required for PDGF to stimulate cell motility, tyrosine phosphorylation and activation of mitogen-activated protein kinase [184]. In that study, the scavenging of H_2_O_2_ with catalase or the use of the antioxidant *N*-acetylcysteine inhibited all the effects of PDGF. As neointimal formation is associated with PDGF signaling, these results support a role for ROS in VSMC migration during vascular injury. An additional *in vitro* study showed that hyperinsulinemia-induced VSMC migration is accompanied by an increase in Nox activity and enhanced mitochondrial production of ROS [185]. In that study, treatment with diphenylene iodonium (DPI) to inhibit ROS production diminished VSMC motility, which suggests that ROS are involved in diabetes-related VSMC migration. Further support for a role of ROS in VSMC migration associated with vascular remodeling comes from a recent study in which antioxidant treatment decreased the motility of VSMCs isolated from mice treated with angiotensin II [141]. Inhibition of c-Src signaling has also been shown to cause ROS suppression and a subsequent reduction in the motility of VSMCs isolated from a testosterone-infused SHR model of hypertension that has increased Nox1 and Nox4 activity [73]. Other evidence for a role of Nox1 in the modulation of VSMC motility comes from a study in Nox1 knockout mice that showed Nox1 is necessary for the phosphorylation of ERK1/2, transactivation of epidermal growth factor receptors (EGFR) and activation of MMP-9. In turn, MMP-9 is responsible for the shedding of *N*-cadherins and the increase VSMC motility observed upon thrombin stimulation [186].

In the microcirculation little is known about ROS-induced VSMC migration, although a number of vascular remodeling processes, such as arterialization and inward remodeling, are likely to involve VSMC movement. Arterialization occurs in processes such as collateral formation, when a capillary, arteriole or small artery is enlarged to increase blood flow to a tissue that has become ischemic due to an arterial blockade. During collateralization it has been shown that VSMCs both migrate and proliferate to increase the thickness of the medial layer and allow for enlargement of vascular diameter and normalization of circumferential stress [187,188,189]. In addition to arterialization, evidence suggests that VSMC migration also occurs during the process of inward remodeling in resistance arteries [155]. During prolonged vasoconstriction we have shown that VSMCs move and change their position within the wall of arterioles that remodeled inwardly. ROS are likely involved in this process as vasoconstriction-induced inward remodeling has been shown to be blocked by either scavengers of ROS, or inhibitors of ROS production [62]. Inward remodeling is also blocked by inhibition of the pathways associated with activation of the small GTPases Rho and Rac [15]. Interestingly, these GTPases are regulated by ROS and associated with its production. The precise role of ROS in mediating VSMC movements in arterialization or inward remodeling requires further investigation to determine whether the mechanisms reported for ROS-mediated VSMC migration elsewhere apply to the microcirculation [10,190].

### 4.4. Reactive Oxygen Species and the Actin Cytoskeleton

Resistance vessels are able to modulate their diameters via the constriction/relaxation of smooth muscle cells within the vessel wall. Contractile stimuli leads to cross bridge cycling between actin and myosin filaments, which shortens smooth muscle cells, thus generating tension that contracts the vessel. In contrast, vasodilatory stimuli inhibit the myosin filaments from interacting with the actin cytoskeleton, which in turn causes smooth muscle cells to lengthen and increase the vessel’s luminal diameter. Historically, the role of the actin cytoskeleton during the contractile process was thought to be static; the filamentous actin that comprises the actin cytoskeleton was believed to be a stable structure that remained relatively unchanged and provided a fixed scaffolding for the myosin filaments to move against and generate tension. A number of studies have challenged this notion, and it is now emerging that the actin cytoskeleton is a dynamic structure that does indeed undergo rapid reorganization, in part via filamentous (F)-actin polymerization, in response to contractile stimuli [191,192,193,194,195,196].

We have observed that reorganization of the actin cytoskeleton also occurs during inward eutrophic remodeling in resistance vessels [15,195]. Following a 4 h exposure of isolated arterioles to constriction agonists, actin polymerization promoted inward remodeling through processes that require ROS generation. Inhibition of either actin polymerization via the addition of cytochalasin D, or ROS generation with apocynin, was sufficient to block the remodeling process [15,62]. Together these observations have led us to hypothesize that one of the roles of ROS during the inward remodeling process is to facilitate actin polymerization/reorganization.

It has been demonstrated that ROS can increase the ratio of F-actin to total actin. In post-hypoxic endothelial cells, the reintroduction of oxygen generates ROS and increases the pool of F-actin. This increase is attenuated by the overexpression of SOD, suggesting that superoxide promotes actin polymerization [197,198]. In addition, Moldovan *et al.* [199], have shown in a wound healing model of endothelial cells that the rate of actin monomer incorporation into F-actin was greater in areas with high levels of measured superoxide, and that this incorporation of monomers was blocked in the presence of either the superoxide dismutase mimetic, manganese (III) tetrakis(1-methyl-4-pyridyl)porphyrin (MnTMPyP), or the inhibitor of ROS generation, DPI. Together, these studies indicate that in specific cell types, ROS increases the rate of actin polymerization thereby increasing the proportion of F- to total-actin. It remains to be determined whether VSMCs *in vivo* undergo a similar response to ROS *vis a vis* the actin cytoskeleton. However, our observation that inward remodeling in response to vasoconstrictor stimuli requires both the production of ROS and the polymerization of actin supports a model in which ROS facilitate reorganization of the underlying cytoskeletal architecture of VSMCs.

Actin polymerization involves the assembly of G-actin monomers into filaments (F-actin) that are characterized by a fast growing barbed end and a slower growing pointed end. The rate-limiting step in this process is the nucleation of monomers into stable oligomers. Kinetically, these reactions are not favored due to the inherent instability of actin dimers. Three classes of actin nucleating proteins have been identified that function to counteract the kinetically unfavorable conditions associated with nucleation: Actin related protein 2/actin related protein 3 (Arp2/3) complex, formins, and tandem monomer-binding nucleators (for review see [200]). In smooth muscle cells, the most studied nucleator is the Arp2/3 complex. It is a seven-subunit complex that exerts its activity predominantly by binding to existing filaments and forming new actin branches. Its polymerizing activity is greatly enhanced via association with a number of nucleation promotion factors (NPF). These function by binding to and activating Arp2/3 as well as recruiting G-actin monomers to the nucleating complex. Thus nucleation is promoted by NPF activation of Arp2/3 and by increasing the local G-actin concentration. The NPF, neuronal Wiskott-Aldrich Syndrome protein (nWASP), has been shown to play an important role in smooth muscle actin polymerization. Inhibition of nWASP, by expressing a dominant negative mutant, blocks actin polymerization in response to contractile stimulation in tracheal smooth muscle cells [201]. In addition, the adaptor protein, CrkII, also plays a role in Arp2/3 mediated actin polymerization by forming a multi-protein complex with Arp2/3 and nWASP that in turn facilitates polymerization. Mutants of CrkII, that failed to associate with nWASP, inhibit force generation and actin polymerization in response to contractile agonists in tracheal smooth muscle cells [202]. Though it has been well established in tracheal smooth muscle cells that Arp2/3 activation increases actin polymerization, there are a limited number of studies in vessels and VSMCs corroborating these results and their relationship with ROS. In mesenteric arterioles, inhibition of CrkII interactions with nWASP and Arp2/3 attenuates the increase in actin polymerization induced by contractile stimulation [203]. In isolated VSMCs, exposure to H_2_O_2_ up-regulates ARP2C (a component of Arp2/3) and down-regulates the actin depolymerizing proteins cofilin and destrin [204]. This presumably favors actin polymerization, but actin polymerization was not assessed in that study. Also in VSMCs, exposure to phorbol esters induces the localization of Arp2/3 complexes to microdomains associated with actin polymerization and newly formed podosomes [205]. This evidence suggests that ROS are involved in actin polymerization and cell migration events in vascular tissues and support our hypothesis that ROS-mediated cytoskeletal modifications are involved in the inward remodeling process associated with hypertension and other cardiovascular morbidities.

The evidence for ROS directly modulating actin nucleation and assembly *in vivo* is not well characterized in the literature. Standard cell free protocols investigating actin polymerization include reducing agents/antioxidants, such as dithiothreitol (DTT) or *beta*-mercaptoethanol, to stabilize nucleating actin oligomers, thereby facilitating the polymerizing process. Additional cell free studies indicate that ROS can oxidize actin and decrease the rate of polymerization [206,207]. However, in cell cultures it would appear that the effects of ROS on polymerization are cell-type specific as well as concentration dependent. It was demonstrated that H_2_O_2_ induces F-actin fragmentation and depolymerization in fibroblasts, while the same concentration of H_2_O_2_ (100–250 μm) increases actin stress fibers in vascular endothelial cells [208]. A much lower concentration of hydrogen peroxide (1 μM) exposure increased actin stress fiber formation in serum-starved fibroblasts [209]. Moreover, as previously indicated, Moldovan and Crawford demonstrated a positive effect of ROS on actin polymerization in vascular endothelial cells. Thus, in the context of vascular remodeling, the *in vivo* effects of ROS on actin polymerization in VSMCs need further study. Though the direct effect of ROS on actin polymerization *in vivo* is unclear, there is evidence that ROS can facilitate filament assembly via activation of signaling cascade(s) that modulate actin nucleating factors and complexes.

The members of the Rho family of GTPases are central regulators of a number of cellular processes associated with vascular remodeling including cell-cell interactions, cell motility, polarity, morphogenesis, and cellular interactions with the ECM. They modulate these processes, in part, through reorganization of the actin cytoskeleton. Within the Rho family, Rho, Rac and Cdc42 are closely associated with remodeling of the actin cytoskeleton. Typical of GTPases, they are activated upon exchange of guanosine diphosphate (GDP) for GTP. In their GTP bound conformation, they interact with downstream effector proteins that transmit activating stimuli, both internal and external, into a cellular response that effects both actin polymerization as well as the bundling of actin filaments into stress fibers. For a general review of Rho GTPases see [210].

Both Rac and Cdc42 have been implicated in facilitating actin polymerization through Arp2/3 activation [211,212,213], whereas Rho appears to initiate elongation of existing filaments via the activation of formins [214,215]. A number of studies have corroborated the role of GTPases in facilitating actin polymerization and/or actin remodeling in smooth muscle cells. Inhibition of Cdc42 blocked tension development and actin polymerization in airway smooth muscle cells exposed to acetylcholine [216]. In an investigation examining VSMC hypertrophy, it was demonstrated that exposure to Leptin increased the F to G-actin ratio in isolated rat portal vein strips, and this coincided with an increase in the activation of RhoA. We have demonstrated that inward remodeling of resistance vessels requires actin polymerization, and that blocking RhoA or Rac1 pathways attenuates remodeling [15].

A small number of studies have demonstrated that ROS can positively modulate the activity of GTPases and their downstream targets in VSMCs. ROS generated by a xanthine-xanthine oxidase mixture contracted rat aortic rings and translocated Rho to the membrane, indicative of Rho activation. This contraction was attenuated in the presence of the Rho kinase inhibitor, Y-27632 [217]. In a rat model of hypertension, pulmonary arteries had elevated basal levels of ROS and activated RhoA compared to control animals. Treatment with the antioxidant, tiron, decreased the level of activated RhoA to that of control animals [218]. In human omental arteries, RhoA activity was increased 3-fold in response to ROS treatment [219].

Cdc42 is negatively regulated by Cdc42GAP (GTPase activating protein), which enhances the GTP hydrolysis of Cdc42, thereby inactivating it. In smooth muscle cells, Cdc42GAP activity is inhibited by exogenously added H_2_O_2_ as well as endogenously produced ROS generated in response to exposure to contractile agonists. Inhibitors of ROS restore Cdc42GAP activity, thereby down-regulating Cdc42 activity in smooth muscle cells exposed to vasoconstrictor agonists [220,221].

These results support a model in which ROS generated by vasoconstrictor stimuli facilitates tension development and arterial remodeling by inducing an increase in actin polymerization in VSMCs. The available data suggests that the rate of actin monomer incorporation is enhanced in response to ROS, though it would appear that this is a consequence of ROS upregulating the activity of positive regulators and/or down regulating inhibitors of actin polymerization, which offsets the negative direct effect ROS has on actin polymerization, via the oxidation of actin.

### 4.5. ROS-Induced Cellular Growth and Apoptosis

In addition to the ROS-induced effects on the phenotypical changes and migration of VSMCs, oxidative stress is also associated with cellular growth and apoptosis [222]. The effects of superoxide anion appear to be mostly mitogenic, whereas those of H_2_O_2_ seem to induce apoptosis [222]. However, there are contradictory results that show exposure of VSMCs to H_2_O_2_ results in increased DNA synthesis, a necessary prerequisite for cell growth [223]. In general, exogenously generated ROS have varying effects on VSMC growth and apoptosis that appear to depend on the origin of the cells and the ROS generating system. In addition, these differential effects of ROS are related to the type of ROS, the concentration of the oxidant, the amount of time the cell is exposed to the oxidant, and the cellular localization targeted by the oxidant. For example, in VSMCs, H_2_O_2_ at high concentrations induces apoptosis [224,225], while at lower concentrations it stimulates growth and differentiation [226]. 

In hypertension-induced remodeling it has been shown that ROS contribute as mediators of VSMC growth [139,227,228]. VSMC hypertrophy and hyperplasia have been observed in the vessels of animals treated with angiotensin II. As mentioned above, angiotensin II is known to activate Nox and increase the production of superoxide anion. Therefore an association appears to exist between angiotensin II stimulation and VSMC hypertrophy/hyperplasia. However, a study in which mice overexpressing human SOD were exposed to angiotensin II suggest the effects of superoxide influence only the pressor response to angiotensin II and not its effects on vascular hypertrophy [229]. Furthermore, evidence from mice overexpressing human catalase suggests that in angiotensin II treated mice, H_2_O_2_ and not superoxide, plays an important role in the hypertrophy of the arterial wall, while its effect on the increase in blood pressure is negligible [230]. Contrary to this, in rat VSMCs superoxide produced by XO appears to have mitogenic effects [222]. In humans, XO derived superoxide does not affect proliferation but increases protein synthesis and hypertrophy of VSMCs [231]. Regardless of the type of ROS involved in the induction of VSMC growth, ROS function as a secondary messenger in the pathways normally associated with cell growth and hypertrophy, such as those including mitogen-activated protein kinase (MAPK), Akt and c-Jun [228,232]. For example, in VSMCs stimulated with angiotensin II, p-38 MAPK is an essential component of ROS mediated hypertrophy [233]. In addition, the ROS-induced hypertrophy/proliferation of murine VSMCs was inhibited by the overexpression of Cu/Zn-SOD or catalase, which diminish the epidermal growth factor (EGF)-induced phosphorylation of ERK1/2 or p-38 MAPK [234]. Overall, data suggest that ROS play an important role in hypertension-induced VSMC hypertrophy and hyperplasia, but the precise mechanisms by which different ROS participate in vascular hypertrophy remain to be fully elucidated.

Apoptosis contributes to the structural changes taking place in the vascular wall under physiological and pathological conditions. In hypertensive mRen2 rats, angiotensin II induced production of ROS is associated with increased apoptosis and vascular remodeling [235]. Inhibition of ROS generation with apocynin blocks the formation of abdominal aortic aneurysm in a murine model by reducing apoptosis of medial cells [236]. Similarly, enhanced VSMC apoptosis has been observed in mesenteric resistance arteries of SHRs [237], as well as in Angiotensin II infused rats [238]. Some of the mechanisms believed to be involved in the apoptosis of VSMCs in hypertension are covered in more detail in [239] and include: activation of angiotensin II receptor type 2 [240] as well as L-type calcium channels [241]. The evidence presented above suggests that ROS play an important role on the dynamic reduction and increase in VSMC number and size in the vascular wall.

### 4.6. ROS-Induced ECM Reorganization

Remodeling of the vasculature also progresses through the degradation, synthesis and reorganization of the ECM in the vascular wall. The most important enzymes associated with ECM reorganization are the MMPs. The MMPs are a group of zinc-dependent endopeptidases that collectively are able to degrade a wide array of ECM proteins such as collagen, elastin, gelatin, and fibronectin. Within the ECM, MMPs are involved in the remodeling of the vessel wall, cleavage of cell surface receptors [242], shedding of precursor signaling molecules [243] and the modulation of chemokine-cytokine signaling [244]. An increased activity of MMPs and subsequent ECM reorganization has been postulated to contribute to vascular remodeling in senescent rats [245,246]. Also, increased circulating levels of MMPs and their endogenous inhibitors (TIMPs) have been found in association with vascular remodeling in human hypertension [247]. Thus it has been suggested that plasma concentrations of MMP-2, MMP-9 and TIMP-1 could serve as markers of cardiovascular remodeling in hypertension or in type 2 diabetic patients where MMP-9 in particular is elevated [248,249]. Similarly, in the SHR, circulating levels of MMP-9 are increased compared with normotensive rats [250], which suggests that in hypertension the activity MMPs is up-regulated and contributing to vascular remodeling. 

Although evidence indicates MMPs are involved in the activation of enzymes that produce ROS, numerous studies have shown that ROS, in particular superoxide and H_2_O_2_, are involved in the increased expression and activation of MMPs in VSMCs [176,186,251,252,253]. An example of the former is that MT1-MMP (a membrane bound MMP) has been shown to be necessary for the activation of Nox in the advanced glycation end products (AGE)-induced ROS increase observed in diabetic VSMCs [254]. In that study, direct AGE receptor (RAGE) interaction with MT1-MMP was shown to be required for the activation of Nox through a pathway involving Rac1/p47phox. Conversely, many examples exist that indicate multiple ROS producing enzymes and oxidant molecules increase the expression and activity of MMPs. XO, for example has been reported to be involved in the activation of MMP-2 [255]. Other studies investigating the role of angiotensin-II-induced MMP activation in VSMCs have shown that MMP activation, specifically that of MMP-1 and MMP-2, occurs downstream of Nox-dependent ROS production [256,257,258]. In animal models of hypertension, results suggest that vascular remodeling is associated with both MMP-dependent activation of ROS producing enzymes and ROS-dependent upregulation and activation of MMPs. This is particularly evident in the murine two-kidney one-clip model (2K-1C) of hypertension, where inhibition of MMP activity with doxycycline reduces blood pressure, ameliorates the endothelial dysfunction associated with reduced levels of NO, and reduces the levels of ROS in addition to reducing MMP activity in conduit arteries [259]. In the same model of hypertension, the inhibition of NF κB resulted in the reduction of the transcription rate of MMP-2, MMP-9 and ROS production [260]. Furthermore, antioxidant treatment with tempol, in 2K-1C mice, reduced MMP-2 activity and attenuated vascular remodeling suggesting a role for superoxide in this process [261]. These studies reveal that at the level of conduit arteries MMPs play an important role in the remodeling of the vessel wall, and that ROS, in most cases, are responsible for inducing their increased activity.

Interestingly, only a small number of studies have investigated the role of MMPs in microvascular remodeling. In one study performed in rat mesenteric arteries, the maintenance of constriction, by angiotensin-II or phenylephrine, appears to require MMP-2 and MMP-7 to transactivate EGFR and maintain tone through PI3K dependent signaling [253]. The transactivation of EGFR in turn also increases the production of ROS. Also the remodeling process induced by altered flow in mesentery resistance arteries has been associated with inflammation and increased MMP-1 and MMP-9 activity [142]. The inflammatory process facilitates remodeling while the type of remodeling, inward or outward, appears to be determined by the hemodynamic conditions present in the vessel (low flow or high flow) [142]. Recently, we have shown that ROS are involved in mediating MMP activation during the process of vasoconstriction-induced resistance vessel remodeling [62]. Our study demonstrated that MMP activity was increased and necessary to induce inward eutrophic remodeling in vessels constricted with norepinephrine and angiotensin-II. ROS inhibition prevented MMP activation and inward remodeling, suggesting that in vasoconstriction-induced inward remodeling, ROS signaling is needed for MMP activation. The source of ROS and MMPs in our study appeared to be VSMCs and fibroblasts present within the vascular wall. In comparison, the source of MMPs involved in high-flow induced outward remodeling is likely macrophages residing in the vascular wall or adjacent to the blood vessel.

### 4.7. Reactive Oxygen Species Contribution to Rarefaction

Rarefaction is defined as the disappearance of capillaries and pre-capillary arterioles in a vascular bed that results in a reduced spatial density of microvascular networks [262]. Rarefaction is one of the most common findings in clinical and experimental hypertension. The density of capillaries can decrease through vessel destruction and/or deficient angiogenesis. The current paradigm indicates that during rarefaction the endothelium suffers a series of functional changes that are associated with decreased vascular relaxation and result in microvessel constriction, loss of blood perfusion and eventual disappearance of capillaries. Recent data suggest that abnormal endothelial apoptosis causes rarefaction [263,264]. The exact mechanism of enhanced endothelial apoptosis is still under investigation, but *in vitro* experiments with vascular endothelial cells suggest that during rarefaction, ROS may induce apoptotic cell death through Nox activation and mitochondrial dysfunction [125,265,266]. In support of the above statement, oxidative stress has been shown to be involved in the promotion of rarefaction through endothelial apoptosis in hypertensive rats, while treatment with antioxidants has resulted in a reduction of microvessel loss [267,268]. Similarly, in the SHR the addition of tempol or apocynin reversed the impaired collateral growth associated with hypertension, suggesting that ROS dampen collateral formation in hypertension [269]. Although activation of the Noxs contributes significantly to the endothelial dysfunction leading to rarefaction, it appears that not all Nox enzymes have deleterious effects. Recently, in a model of kidney injury, rarefaction and oxidative stress were shown to increase following Nox4 silencing, but not after Nox2 silencing [270]. This suggests that Nox4, which is the major Nox isoform in renal tissues, has a protective effect in the kidney.

The angiogenesis responsible for the formation of new microvessels that counterbalances rarefaction appears to be impaired in the normotensive offspring of individuals with essential hypertension [271,272]. Impaired angiogenesis is also present in animal models of aging [273], where this phenomenon is associated with reduced expression of pro-angiogenic factors and receptors [274,275,276,277,278]. Furthermore, in diabetes, which is associated with severe peripheral impairment of angiogenesis, there is increased production of ROS through mechanisms involving the Noxs and XO [279,280]. Also in diabetes, the inhibition of eNOS uncoupling or the addition of BH_4_ results in decreased superoxide production, suggesting that angiogenesis impairment in diabetes is associated with a reduction in antioxidant defenses combined with eNOS uncoupling and an increase in ROS production [281]. A few studies have also shown that increased superoxide production, formation of peroxynitrite, as well as reduced NO bioavailability diminish the proangiogenic responses of patients with type 1 or type 2 diabetes [282,283]. This further suggests that ROS production in diabetes affects peripheral angiogenesis through different mechanisms. In contrast, in the retina of diabetic patients, ROS production leads to PKC-dependent or AGE-dependent increases in VEGF expression [284,285]. In the retina of diabetic patients the migration of endothelial cells and the proliferation of smooth muscle cells can also be stimulated by H_2_O_2_ [285]. While increased peripheral angiogenesis would be welcomed, in the retina the increase in angiogenesis is abnormal and promotes diabetic retinopathy. It therefore appears that the role of ROS signaling in the modulation of angiogenesis is beneficial under physiological conditions. However, under certain circumstances, the induction of pathways that promote angiogenesis are overly enhanced by the presence of oxidative stress and their effects are deleterious [286]. An important issue to consider is that ROS can regulate angiogenesis in several distinct ways, including the modulation of cytokine receptors, the modulation of the expression of genes involved in cell survival and the modulation of multiple signaling pathways involved in vessel formation. A more in-depth review covering the multifaceted roles of ROS in angiogenesis can be found in reference [83].

## 5. ROS and the Myogenic Response

As mentioned above, it has been postulated that presence and modulation of the myogenic response in resistance vessels is intrinsically associated with the type and level of remodeling these arteries develop in association with hypertension and other pathophysiological conditions [42,287]. Myogenic vasoconstriction is a vascular response in which increased intravascular pressure results in VSMC activation and contraction. The increase in intravascular pressure is usually associated with VSMC stretch or an increase in tension at the anchoring points of the cell with the ECM or neighboring cells. Therefore evidence for an involvement of ROS in myogenic phenomena comes from studies that show VSMC stretching is associated with ROS production [288,289,290]. Those studies further suggest that the primary sources of ROS associated with VSMC stretching are the Noxs [291]. In whole blood vessels, increased intravascular pressure has also been associated with an increased production of ROS [292,293,294,295,296,297]. This appears to take place both in conduit arteries as well as in myogenically active resistance vessels. The addition of exogenous scavengers of ROS to resistance vessels has been shown to inhibit the myogenic vasoconstriction induced by intravascular pressure increases [298]. This suggests that in myogenically active vessels, ROS produced in response to intravascular pressure elevation is associated with signaling pathways that induce VSMC contraction, whereas in conduit vessels (which are not myogenically active) these pathways may not be present. A recent study suggests that in the myogenically active middle cerebral artery, ROS-dependent pathways associated with myogenic vasoconstriction include oxidation of the phosphatase PTEN and activation of the protein kinase Akt [298]. That study also indicates that mitochondria are the main sources that provide ROS during pressure-induced myogenic vasoconstriction. Because other studies indicate that the Noxs are the main sources of ROS in stretched VSMCs or vessels exposed to increased intraluminal pressure, it remains to be determined whether the sources of ROS associated with myogenic vasoconstriction vary depending on the vascular bed studied and the myogenic properties of the vessel.

Recently, it was discovered that G-protein couple receptors are mechanosensitive and that the angiotensin II type 1a receptor (AT1aR) is required for the mesenteric and renal arteries of the mouse to be myogenically active [299]. Because AT1R activation is associated with Nox activation and ROS production, it is likely that myogenic vasoconstriction in these vessels is associated with superoxide production by the Noxs. We recently discovered that ROS activate integrins in VSMCs stimulated with lysophosphatidic acid (LPA) [300]. Furthermore, we showed that ROS produced by isolated resistance arteries stimulated with LPA increase the level of myogenic vasoconstriction of these vessels in response to sequential increments in intravascular pressure. Because we have previously determined that blockade of α_v_β_3_ and α_5_β_1_ integrins in these vessels inhibits myogenic vasoconstriction [301], it is tempting to speculate that ROS produced subsequent to stretch activation of AT1aR activates integrins that then participate in promoting a number of the VSMC intracellular changes associated with both myogenic vasoconstriction as well as vascular remodeling phenomena.

## 6. Conclusions

All cells present within the wall of microvessels produce ROS. The role of these ROS on vascular pathophysiological processes appear to vary depending on the source of ROS within the cell, the specific cell that produces ROS and the overall amount of bioavailable ROS. The scientific literature consistently indicates that while ROS play important roles in physiological cell signaling pathways, excessive production of ROS or a reduced capacity to scavenge these radicals can damage microvessels and induce pathophysiological remodeling of the microcirculation. The remodeling of microvessels induced by ROS modify the structural characteristics of the vascular wall and may include modifications in cytoskeletal structures, cellular attachments, cell phenotype, apotosis and composition of the ECM. A better understanding of the pathways associated with ROS-induced remodeling of microvessels should provide targets for the development of strategies to manipulate vascular remodeling and ameliorate the detrimental consequences of vascular disease. 
